# Adipokine-Modulated Immunological Homeostasis Shapes the Pathophysiology of Inflammatory Bowel Disease

**DOI:** 10.3390/ijms21249564

**Published:** 2020-12-15

**Authors:** Yi-Wen Tsai, Shin-Huei Fu, Jia-Ling Dong, Ming-Wei Chien, Yu-Wen Liu, Chao-Yuan Hsu, Huey-Kang Sytwu

**Affiliations:** 1 Department of Family Medicine, Chang Gung Memorial Hospital, Keelung, No. 222, Maijin Road, Keelung 204, Taiwan; tsaiyiwen@gmail.com; 2 College of Medicine, Chang-Gung University, No. 259, Wenhua 1st Rd., Guishan Dist., Taoyuan City 333, Taiwan; 3 Graduate Institute of Medical Sciences, National Defense Medical Center, No. 161, Section 6, Min Chuan East Road, Neihu, Taipei 114, Taiwan; 4 Department and Graduate Institute of Microbiology and Immunology, National Defense Medical Center, No. 161, Section 6, Min Chuan East Road, Neihu, Taipei 114, Taiwan; winniefold@gmail.com (S.-H.F.); pantherchien@gmail.com (M.-W.C.); 5 National Institute of Infectious Diseases and Vaccinology, National Health Research Institutes, No. 35, Keyan Road, Zhunan, Miaoli 350, Taiwan; irenejld00@gmail.com (J.-L.D.); candy_77615@yahoo.com.tw (Y.-W.L.); 6 Graduate Institute of Life Sciences, National Defense Medical Center, No. 161, Section 6, Min Chuan East Road, Neihu, Taipei 114, Taiwan; 7 Molecular Cell Biology, Taiwan International Graduate Program, No. 128, Academia Road, Section 2, Nankang, Taipei 115, Taiwan

**Keywords:** inflammatory bowel diseases, adipokines, immunologic homeostasis, metabolic regulation

## Abstract

Inflammatory colon diseases, which are a global health concern, include a variety of gastrointestinal tract disorders, such as inflammatory bowel disease and colon cancer. The pathogenesis of these colon disorders involves immune alterations with the pronounced infiltration of innate and adaptive immune cells into the intestines and the augmented expression of mucosal pro-inflammatory cytokines stimulated by commensal microbiota. Epidemiological studies during the past half century have shown that the proportion of obese people in a population is associated with the incidence and pathogenesis of gastrointestinal tract disorders. The advancement of understanding of the immunological basis of colon disease has shown that adipocyte-derived biologically active substances (adipokines) modulate the role of innate and adaptive immune cells in the progress of intestinal inflammation. The biomedical significance in immunological homeostasis of adipokines, including adiponectin, leptin, apelin and resistin, is clear. In this review, we highlight the existing literature on the effect and contribution of adipokines to the regulation of immunological homeostasis in inflammatory colon diseases and discuss their crucial roles in disease etiology and pathogenesis, as well as the implications of these results for new therapies in these disorders.

## 1. Introduction

### 1.1. The Pathophysiology of Inflammatory Colon Disease

Inflammatory bowel disease (IBD), which includes ulcerative colitis (UC) and Crohn’s disease (CD), is a global health concern because of the burdens of medical costs attributable to their acute and chronic complications and the poor quality of life of patients [1,2]. IBD has a complex etiology and pathophysiologic factors, including genetic variants, an inappropriate inflammatory response to the gut microbiome, dysregulation of immune-modulated intestinal inflammation, and environmental factors [3,4,5]. Although the pathogenesis of IBD is complicated and the clinical presentations of UC and CD are distinct, there are common phenotypes, such as the deregulation of colonic immune response and chronic inflammation, implicated in the pathogenesis of both subtypes [6,7,8]. Therefore, much of the research on IBD pathogenesis and therapeutic investigation has focused on the immune system and its maintenance of homeostasis processes.

During perturbation of the integrity of the intestinal epithelial barrier and the gut lumen defects, microbial translocation occurs, which promotes activation of the innate immune cells such as macrophages and dendritic cells in the lamina propria. Antigen-presenting cells then activate naïve T cells and promote their development into different types of effector CD4^+^ cells, which alter gut homeostasis. The development of pathogenic T cells is linked with the environmental factors correlated with IBD pathology, such as tissue metabolism and the production of pro-inflammatory cytokines. Moreover, the development of chronic colon inflammation occurs when uncontrolled infiltration of inflammatory CD4^+^ T cells into the lamina propria of patients with IBD because of their dysfunctional barrier and defeat in immune tolerance for intestinal antigens [8,9]. Current studies reveal that T helper (Th) cells are major modulators of intestinal colitogenesis [10]. Th cells responding to T-cell receptor-mediated activation by a range of pathogens and the stimulation of cytokines can differentiate into several Th cell lineages, which result in distinct effector subsets, including T Th1, Th2, Th17, and regulatory T cells (Treg), which play important roles in the immune response [11]. Genome-wide association studies have demonstrated that single-nucleotide polymorphisms in genes encoding transcription factors modulate cytokine-mediated regulation of the immune responses correlated with the pathogenesis of IBD [4,12]. The immunological pathogenesis of IBD involves an altered immune response with the pronounced infiltration of adaptive immune cells into the lamina propria of the intestines, and the augmented expression of mucosal pro-inflammatory cytokines, such as tumor necrosis factor (TNF)-α, interferon (IFN)-γ, and the IL-12/IL-23 pathways [9,13,14,15,16].

### 1.2. The Effect of Obesity on the Development of IBD

The incidence of IBD is augmenting in North America, Europe, and Asia, and is speeding in newly industrialized countries [2,17]; the prevalence of overweight and obesity is also growing in parallel to the IBD pandemic [18]. The incidence of IBD and obesity have increased markedly in countries in which the lifestyle changes have included diets rich in animal fat and low in dietary fiber and insufficient physical activity [19,20,21,22]. Obesity significantly involves an expansion of the entire volume of fat tissue accompanied by distinct alterations in the balance of the cellular and humoral immunity [23]. The expression of pro-inflammatory cytokines and the infiltration of adipose tissue by immune cells such as Tregs and macrophages are augmented in obese compared with lean individuals [23]. Moreover, obesity is correlated with a chronic inflammatory state, illustrated by the signaling pathway activation of inflammation, enhanced synthesis of C-reactive protein and pro-inflammatory cytokines, and activation of the pro-inflammatory transcription factors in adipocytes compared with that in healthy controls [24,25]. These changes in adipose tissue and their metabolic and systemic consequences have contributed to the model of obesity as an inflammatory state [26]. This chronic inflammation eventually leads to intestinal inflammatory diseases. Visceral adiposity is particularly correlated with the development of insulin resistance and associates with metabolic syndrome [27]. Observational studies have reported that around 20%–30% of children with IBD are overweight, while prior IBD-related surgery was correlated with obesity in these pediatric patients with CD [28]. The same phenomenon was observed in adult patients with IBD in Tayside Scotland: only a small number of patients with IBD were underweight, whereas 18% of the population with CD were obese and 52% were overweight, demonstrating that the incidence of IBD significantly increased in obese patients [29]. Furthermore, previous studies reported that the development of obesity is critically associated with alterations of in the gut microbiota, which, in turn, impact mucosal immunity and intestinal inflammation [30,31].

The pro-inflammatory state of visceral obesity is associated with several gastrointestinal diseases, such as IBD and fatty liver disease, and a crucial correlation noted between IBD and obesity concerns non-alcoholic fatty liver disease (NAFLD) [32]. It has been reported that the prevalence of NAFLD is augmented among IBD patients as compared to non-IBD controls, and this phenomenon occurs commonly at a young age [33]. Moreover, the diagnosis of NAFLD by transient elastography with controlled attenuation parameters is a common comorbidity for IBD patients and is correlated with extrahepatic diseases [34]. These results suggest that the perpetuation of inflammation promotes risks for co-morbid conditions shared between IBD and NAFLD, and these developments could link the epidemiology of these diseases.

### 1.3. The Interaction of Adipose Tissue and the Intestinal Immune System

Adipose tissue, which was initially considered to be merely an energy store, is now recognized as an endocrine organ that not only stores energy but also secretes or produces various biologically active substances called adipokines [35] and interacts closely with the immune system [36,37]. Thus, adipose tissue is now regarded as an endocrine organ with several functions [38]. Adipose tissue can be separated into subcutaneous and visceral adipose tissue (VAT), the relative amounts of which vary greatly between individuals, and the total body weight ranges from 5% to 60% [39]. Adipose tissue is composed of multiple cell types, of which adipocytes are the most prominent, followed by vascular endothelial cells, macrophages [40,41], and lymphocytes [42,43], which are found in the stromovascular fraction. It has been reported that pediatric patients with CD were observed with higher VAT volumes than healthy controls and the odds of CD-related hospitalization was correlated with the increase in VAT volume [44], suggesting that obesity is a critical risk factor related to the development of CD in pediatric patients; VAT could be a better predictor of disease progression than obesity determined by body mass index (BMI) and is a possible marker of the progress of pro-inflammation or metabolic activity [45,46]. Studies in patients with CD by performing visceral adiposity as the obesity quantity have more consistently shown an augmented risk of complications with CD than those using BMI as the obesity marker [47,48,49]. Computed tomography for mesenteric fat index (MFI) have suggested that the ratio of the area of VAT to that of subcutaneous fat is a marker for the CD progression: VAT area and MFI values were correlated with the postoperative recurrence of CD and the ratio of VAT to subcutaneous adipose tissue was associated more strongly than BMI with the increase in disease activity of CD and its structural behavior [50].

Adipocytes comprise white and brown cells, presumably including diverse intermediate forms. Because white fat cells are the critical part in adipose tissues from adults [51,52], this review focuses on these. Mature white adipocytes consist of a large fat droplet surrounded by a margin of remaining cytoplasm and the nucleus. Adipocytes are characterized by their cellular plasticity, store the body’s energy supplies, and actively secrete various adipokines [53]. Macrophages and T cells are the two most common immune cells present in adipose tissue. Altered infiltration of immune cells and the dysregulated production of pro-inflammatory cytokines have been documented in obesity [41,54,55,56]. For example, adipose tissue-resident T cells increase approximately threefold in the high-fat diet (HFD) mouse model of obesity [57]. The adipokines comprise a group of mediators secreted by adipose tissue, including adiponectin, leptin, apelin, and resistin. Recent evidence indicates that adipokines can modulate immune functions in addition to their regulation of metabolic homeostasis within adipose tissue [25,58]. For example, previous studies have demonstrated that adiponectin can suppress the phagocytic activity of mature macrophages mediated by the complement C1q receptor and inhibit TNF-α production stimulated by lipopolysaccharide (LPS) [36]. In addition, leptin has been reported to be a T-cell immune response modulator that can enhance the cytotoxicity of natural killer (NK) cells, induce activation of granulocytes, macrophages and dendritic cells, and polarize Th cell subsets differentiation toward a pro-inflammatory Th1 phenotype rather than an anti-inflammatory Th2 phenotype [37,59]. Thus, investigation of the adipokine modulation of immune responses as a potential therapeutic target to modulate inflammation in the context of the pathophysiology of IBD may be a focus of future research in inflammatory colon diseases.

**Figure 1:**
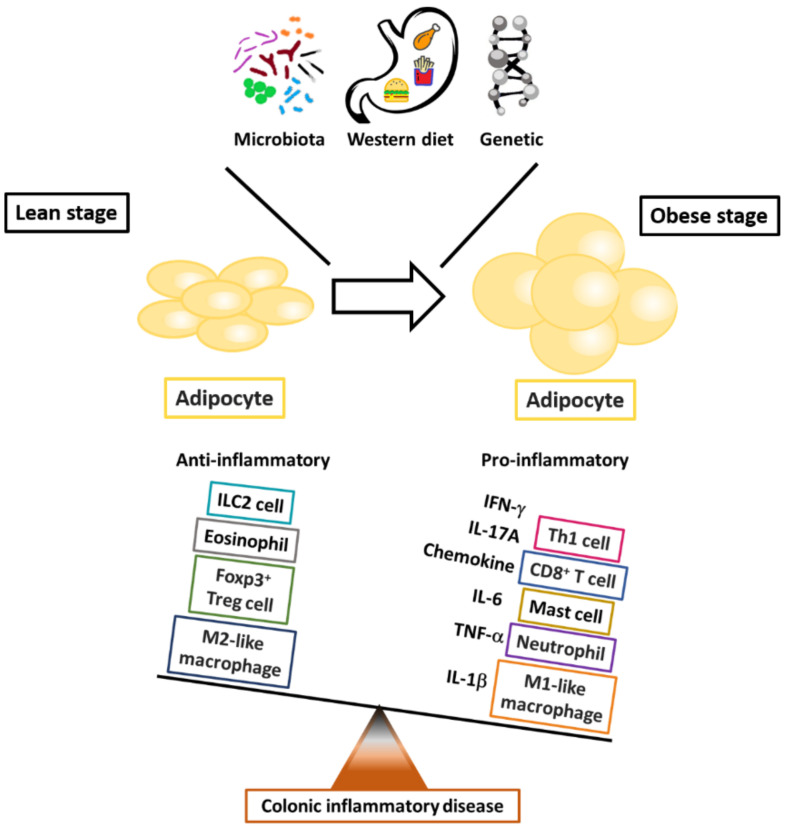
The critical roles of immune cells in fat-associated colonic inflammation. The outcome of obesity-modulated inflammation in adipose tissue reflects the balance between pro- and anti-inflammatory mediators in the systems of innate and adaptive immune cells.

Evidence has demonstrated that excessive adipose tissue accumulation, which is characterized by a chronic inflammatory state involving the activation of pro-inflammatory signaling pathways and increased pro-inflammatory cytokine production [24], increases the risks of developing many chronic diseases, including UC and CD [24,60,61,62,63]. Recent reports have demonstrated a particular role of mesenteric adipose tissue that migrates around intestine, so-called “creeping fat”. The presence of creeping fat, in which the expression of peroxisome proliferator-activated receptor (PPAR)-γ and TNF-α is augmented [64], possesses an association with transmural inflammation, fibrosis, and stricture formation [65], which lead to pathogenic intestinal inflammation in patients with CD [45,66]. Moreover, recent epidemiological analyses have demonstrated the influence of the Western diet on changes in body composition and aggravation of colitis severity in patients with IBD [20]. Thus, both in vitro and in vivo studies and epidemiologic research indicate the potential role in inflammatory colon diseases of adipokine-modulated immune regulation of the interplay between adipose tissue, chronic inflammation, and immune cells (Figure 1). This review focuses on studies that relate immune cells, adipose tissue, and the selected adipokine-modulated production of pro-inflammatory cytokines to immunologic homeostasis in inflammatory colon diseases.

## 2. Adipokine-Modulated Immunological Regulation in the Pathophysiology of Inflammatory Colon Disorders

### 2.1. Obesity and Alteration of Immune Responses in Adipose Tissue

The major contributors to the adipose tissue secretome are tissue-resident immune cells. Accumulating studies have demonstrated distinct differences in the immune cell recruitment around adipocytes and the distinct production and regulation of adipokines in the obese and the lean states. In vivo, these adipose tissue-resident immune cells display plasticity and are able to undergo a phenotypic switch for changes in the local microenvironmental stimuli [57,67]. Neutrophils, one of the first responders of the innate immune response that are recruited to adipose tissue after initiated HFD feeding in vivo, produce TNF-α to inhibit insulin signaling [68]. In the adaptive immune system, adipose tissue-resident macrophages (ATMs) and potentially adipocytes themselves are capable of presenting antigens on the MHC class II complex to T cells or B cells to induce the subsequent adaptive immune response. T cells can be further categorized according to their profile of functional characteristics.

#### 2.1.1. Macrophages

ATMs are composed of two broad classes, such as classically activated M1-like ATMs and alternatively activated M2-like ATMs, based on the landscape of genetic characteristics [40,69,70]. In the pro-inflammatory obese state, the hypertrophy and apoptosis of adipocytes induce cellular stress. The adipocytes secrete pro-inflammatory factors, including saturated fatty acids, leukotriene B4, and IFN-γ [71], which stimulate the recruitment of monocytes to the fat tissue, where they can further polarize into pro-inflammatory M1-like macrophages in response to inflammatory insults [41]. By contrast, in the lean state, anti-inflammatory M2-like ATMs are elicited by the type-2 cytokine IL-4, which is produced at high quantities in lean adipose tissue [72]. It has been reported that the roles of ATMs during the inflammatory progress of adipose tissue is critical for the metabolic maintenance of systemic insulin sensitivity [73,74]. Moreover, the accumulation of M1-like ATMs with enhanced levels of pro-inflammatory cytokines such as TNF-α and IL-1β is correlated with the obesity-linked metabolic regulation, whereas the remodeling of tissues and the improvement of inflammation in fat are positively associated with the development of M2-like ATMs [75]. The expression PPAR-γ [76] and PPAR-δ [55], which are essential for maintenance of the alternatively activated state of ATMs, are augmented under the IL-4 stimulation. IL-5 and IL-13, produced by innate lymphoid cells type 2, augment the maturation and activation of eosinophils. The adipose tissue-resident eosinophils and Treg cells secrete IL-4 and IL-10 to help maintain the alternative activation of macrophages. The adipocyte-mediated adiponectin also promotes the preservation of alternatively activated macrophages, which supports the insulin sensitivity of adipocytes [57].

#### 2.1.2. Effector T Helper Cells

In the context of obesity, VAT-resident Th1 cells and Th17 cells are pro-inflammatory, whereas Th2 cells and Treg cells have anti-inflammatory properties. The number of adipose tissue-resident Th1 cells, which contribute to the activation of M1-like ATMs and adipose tissue inflammation by expression of IFN-γ and upregulation of *T-box 21* (T-bet) gene expression, increases in obesity, whereas the richness of Th2 and Treg cells is decreased [56,77]. The secretion of pro-inflammatory cytokines and chemokines by adipose tissue-resident immune cells, such as effector CD8^+^ T cells and Th1 cells, constitutes a network of immune cell recruitment, further contributing to a chronic inflammatory state.

The inflammatory reaction of hypertrophic mesenteric adipose tissue in patients with CD is characterized by increased expression of PPAR-γ and TNF-α within the mesenteric fat depot [64,78]. Lord et al. demonstrated that leptin induced T-cell proliferation and resulted in enhancing Th1 cytokines and suppressing the expression of Th2 cytokines, revealing that the cytokine production of Th1 or Th2 cells from naive CD4^+^ T cells under in vitro polarizing conditions was abrogated in cells from the deficiency of leptin *ob/ob* mice compared with wild-type mice [79]. In an in vivo study using an oxazolone-induced colitis model, leptin-deficient *ob*/*ob* mice were protected from colitis by suppressing expression of the key transcription factors such as T-bet and GATA-3 under Th1 and Th2 polarization. In addition, recent evidence has shown that leptin administration induced inflammation in a TNF-α-dependent manner by exerting diverse pro-inflammatory effects on immune cell differentiation and function in a patient with combined acquired generalized lipodystrophy and CD [80]. In 2005, Mucida et al. [81] used a T-cell specific leptin receptor-deficient mouse model to demonstrate impaired Th17 differentiation because of decreased activation of the signal transducer and activator of transcription 3 (Stat3) cascades and expression of RAR-related orphan receptor γt (RORγt), as well as a decrease in IL-17/IFN-γ secretion in vitro and in vivo. By contrast, in a transfer model, host *Rag1^−/−^* mice were protected from colitis by receiving LR-deficient T cells. These findings implied that LR signaling is essential and required for Th17 differentiation.

#### 2.1.3. Regulatory T Helper Cells

**Figure 2:**
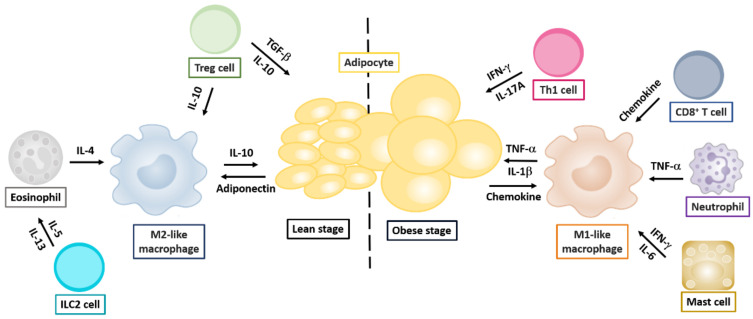
Cytokine-modulated regulation of adipose tissues by innate and adaptive immune cells. Cytokine networks in the lean or obese stage influencing the development of innate and adaptive immune cells are indicated. Cytokine production by distinct T helper cells is indicated in components involved in regulating Th1, Th2, Th17, and Treg cells.

Treg cells expressing the transcription factor FoxP3 are the master regulators of the immune system, maintaining homeostasis and peripheral immune tolerance and preventing autoimmunity. Studies in animal models have shown that with increasing age, an increased percentage of Treg cells accumulate in VAT, representing up to 80% CD4^+^ T-cell population in lean C57BL/6 mice, compared with an average of the 20% CD4^+^ T cell population in lymphoid organs. However, a significant reduction in Treg cell numbers was observed in adipose tissue from animal models of insulin-resistant obesity [56]. These VAT Tregs can control inflammation and metabolism. Accumulated evidence has suggested that the VAT Tregs have a particular phenotype and transcriptome different from Treg populations in the lymphoid-organs [82]. VAT Treg cells produce cytokines that differentially promote the inflammatory factor synthesis and glucose uptake by adipocytes [83]. For example, the gene transcripts upregulated in VAT Treg cells compared with VAT-resident conventional T cells included molecules involved in the migration of leukocytes, such as CCR9 and CXCL10, while other molecules, such as CCL5 and CXCR3, were decreased in VAT Treg cells. An extreme elevation of IL-10 transcript levels was also reported in VAT Treg cells [56]. In addition, Vasanthakumar et al. [84] demonstrated that sex hormones, such as androgen, also regulated VAT inflammation and facilitated the recruitment of Treg cells in a CCL2–CCR2 axis-dependent manner. It has also been shown that leptin can inhibit Treg proliferation: in both *ob/ob* and *db/db* mouse models, an increase in the proliferation of functional Treg cells was observed in the absence of leptin [85]. Likewise, Reis et al. [86] reported a higher frequency of Tregs under steady-state conditions in T-cell specific leptin receptor-deficient mice. In an in vivo IL-10-deficient mouse model, treatment with a pegylated leptin antagonist resulted in attenuation of the clinical colitis score, a reversal of colitis-associated pathogenesis suppression of systemic and mucosal inflammatory cytokine production, and an increase in mucosal Treg cells. Taken together, these studies reveal that both the innate and adaptive immune cells play an active part in the modulation of the adipose tissue and inflammatory responses (Figure 2), implying that harnessing the anti-inflammatory properties of VAT Treg cells to modulate the immune and metabolic function may have potential therapeutic approaches.

### 2.2. Adipokines and the Pathogenesis of Inflammatory Colon Diseases

#### 2.2.1. Adiponectin

Adiponectin, composed of 244 amino acid residues, is among the adipokines that can regulate insulin sensitivity, glucose, and lipid metabolism, and was originally regarded as predisposing to anti-inflammation in atherosclerosis [36,87,88]. Accumulating evidence has shown that adiponectin can also regulate the adaptive humoral immune response [36,89]. One in vitro study demonstrated that adiponectin could suppress TNF-α–induced phosphorylation of IκBα and subsequent activation of nuclear factor (NF)-κB in human aortic endothelial cells [90]. Another in vitro study using cultured porcine macrophages found that adiponectin can inhibit phagocytic activity and the production of pro-inflammatory cytokines, such as TNF-α and IL-6, by suppressing NF-κB signaling and the activity of extracellular signal-regulated kinase 1/2 (ERK1/2) [89]. An ex vivo study demonstrated that adiponectin can selectively augment the tissue inhibitor of metalloproteinases-1 expression in human monocyte-derived macrophages through the induction of IL-10, revealing an interaction of adiponectin/IL-10 to combat vascular inflammation [91]. Collectively, these results suggest that adiponectin can promote the anti-inflammatory response through the modulation of phagocytosis or suppression of inflammatory cytokine production by macrophages.

Treatment of mice with dextran sodium sulfate (DSS) or trinitrobenzene sulfonate (TNBS) is two common methods to induce experimental colitis by disruption of intestinal epithelial cell proliferation and production of pro-inflammatory mediators in the colonic mucosa. To date, the role of adiponectin in intestinal inflammatory disease is considered controversial; some in vivo studies have demonstrated that adiponectin exerts the pro-proliferative or pro-inflammatory influence through the activation of ERK, p38 mitogen-activated protein kinase (MAPK), and NF-κB signaling in colonic epithelial cells [92]. Fayad et al. also revealed that adiponectin-deficient mice are protected from TNBS-induced colitis, whereas the administration of adiponectin restores inflammation via augmented expression of pro-inflammatory cytokines, such as IL-6 and CXCL2, in the colon [93]. By contrast, the further study reported a protective effect of adiponectin in chronic inflammation by using DSS treatment of adiponectin-deficient mice [94]. These findings reveal that acute DSS- and TNBS-induced colitis were ameliorated in a conventional adiponectin-deficient mouse model. In human studies, upregulation of adiponectin levels has been demonstrated in the creeping fat of patients with CD, compared with that in the non-creeping fat of patients with CD or UC and healthy controls [95]. Some in vivo studies have revealed that the role of adiponectin in intestinal inflammatory disease is pro-proliferative and pro-inflammatory through the activation of ERK, MAPK, and NF-κB signaling in colonic epithelial cells [96]. These findings were in contrast to those of several in vitro studies showing adiponectin-modulated anti-inflammatory effects in macrophages. Though the overall impact of adiponectin on acute intestinal inflammation in mouse models remains uncertain, possibly because of the different concentrations of DSS and TNBS chosen for these experimental animal models of colitis, these findings support the concept that adiponectin derived from adipocytes plays an important role in maintaining intestinal homeostasis.

#### 2.2.2. Leptin

The adipokine leptin, which is encoded by the obese (*ob*) gene, is produced by adipose tissue and has a key role in appetite regulation and energy homeostasis [94]. The circulating level of leptin in an individual is proportional to their fat mass. Studies have shown that in animals, the deficiency of either leptin (*ob/ob*) or its receptor (*db/db*) contributes to severe obesity and metabolic dysregulation. Leptin receptor (LR) is widely distributed throughout the body and is highly expressed in the T lymphocytes, colonic epithelial cells, and vascular endothelial cells [97]. Leptin is known to be a pro-inflammatory cytokine that can also modulate the production of various immune cytokines through LR located on the immune cell surface, including T cells, B cells, dendritic cells, neutrophils, and NK cells [98,99,100].

With respect to the effect of leptin on T-cell responses, it has been shown that the long isoform expression of LR on the surface of peripheral CD4^+^ T cells is much higher than that on the surface of CD8^+^ T cells [101]. An in vitro study showed that leptin treatment could lead to augmented Th1 responses with elevated IFN-γ and IL-2 levels and suppression of Th2 cytokine production, which implied that leptin may modulate T-cell responses toward a pro-inflammatory phenotype (Th1 response). In addition, a further study revealed that the treatment of leptin can reverse the suppressive effects of acute starvation by inducing the secretion of large amounts of IFN-γ and suppressing IL-4 production by T cells in a dose-dependent manner [37]. However, the in vivo immunomodulatory potential of leptin mediated directly via CD4^+^ T cells remained to be explored. Several research groups have demonstrated consistent findings of reduced development of thymic T cells, impaired T-cells activation upon stimulation, and impaired effector T-cell responses in leptin-deficient (*ob/ob*) mice [102]. Yu et al. [103] demonstrated reduced Th17 cell frequency in leptin-deficient *ob/ob* mice and showed that the in vitro addition of leptin to the splenocytes and peripheral blood mononuclear cells from *ob/ob* mice can restore the population of Th17 cells to levels comparable to those found in wild-type mice by inducing RORγt transcription and upregulated phosphorylation of Stat3. These findings imply that leptin possesses a strong pro-inflammatory effect by biasing the immune system toward a Th1/Th17 phenotype, and that it is released for the response to inflammatory stimulation, such as bacterial infection, LPS, or IL-6 [104]. Leptin also possesses an enhancing modulatory effect on the proliferation of T cells, and LR is critical for the CD4^+^ T cell differentiation into Th17 cells both in vitro and in vivo [81].

Because LR is expressed on the T lymphocytes that modulate chronic intestinal inflammation in mice [105], several research groups have focused on this area. Leptin mRNA overexpressed in the mesenteric adipose tissue of patients with IBD (both CD and UC) compared with that in healthy controls [106,107]. In addition, the plasma concentration of leptin was increased in several experimental animal models of colitis such as TNBS-mediated colitis and indomethacin-induced ileitis, and was associated with disease severity [108]. Siegmund et al. [79] also demonstrated that the severity of intestinal inflammation in both DSS- and TNBS-induced experimental colitis was significantly attenuated in *ob/ob* mice. These findings identified leptin as a crucial mediator of intestinal inflammation in the leptin-deficient *ob/ob* mouse model because of its ability to reduce the production of pro-inflammatory cytokines, alter T-cell activation, and reduce the phosphorylation of Stat-3, which results in resistance to DSS- or TNBS-mediated intestinal inflammation. In human patients with IBD, increased leptin levels were observed to be associated with UC [109,110]. Sitaraman et al. also demonstrated that luminal leptin acts on colonic epithelial cells as a pro-inflammatory cytokine that induces epithelial damage and neutrophil infiltration that are characteristic histological findings in acute intestinal inflammation in patients with IBD. Upregulation of leptin expression was also observed in inflamed colonic cells, and gut luminal leptin induced activation of NF-κB, the activation of which has been implicated in the pathogenesis of IBD [111]. These findings suggest a new pathophysiological role for colonic epithelial leptin in inflammatory colon diseases.

#### 2.2.3. Apelin

Apelin, which binds to the apelin (APJ) receptor and regulates lipid metabolism and angiogenesis, is a newly recognized adipokine produced from isolated adipocytes [112,113]. The production of apelin in colonic tissues is clearly increased in patients with UC and CD [114]. Synthetic apelin treatment to mice during the development of DSS-induced colitis significantly enhanced the proliferation of colonic epithelial cells, suggesting that enhanced apelin expression in the intestinal recovery stage may result in the repair of the intestinal epithelium in rodent colitis models and in patients with IBD [114]. Recognized as the APJ endogenous ligand, apelin has also been reported to play crucial roles in the modulation of energy metabolism [115]. Further studies have revealed the critical role of apelin signaling in the polarization of endothelial cells [116] and the essential process of lymphatic endothelial cell migration [117]. The apelin/APJ system inhibits inflammation by enhancing lymphatic function [118]. Apelin is highly produced in the adipose tissue of patients with CD compared with that from controls, and the systemic delivery of apelin significantly ameliorates the disease activity index and colitis scores in IL-10-deficient mice by downregulating inflammatory cytokines such as TNF-α and IL-6, suggesting that apelin is a key mediator and a therapeutic target of immune-mediated injury [119]. These results demonstrated that apelin has a supportive role in intestinal lymphatic drainage of IBD.

#### 2.2.4. Resistin

Resistin, which was originally regarded as an adipocyte-associated hormone, is a unique adipocyte-derived cysteine-rich signaling molecule, although it is mostly produced from macrophages and is involved in the cell signaling of autocrine and paracrine types [120]. Resistin exerts a strong pro-inflammatory action in the pathogenesis of murine obesity and diabetes [121]. Resistin is associated with the processes of inflammation because its expression is promoted by pro-inflammatory cytokines such as TNF-α and IL-6 in adipose tissue [122], and it plays critical roles in the pathogenesis of obesity and insulin resistance [123]. The serum levels of resistin are raised in inflammatory states such as IBD [124,125]. Monoclonal antibody therapy against TNF-α significantly reduces serum levels of resistin in patients with IBD [125]. Moreover, resistin also upregulates the expression of pro-inflammatory cytokines such as TNF-α and IL-6 in human peripheral blood mononuclear cells [126].

#### 2.2.5. Discussion

Adiponectin, one of the adipokines produced by adipocytes, is regarded as anti-inflammatory adipokine in non-IBD patients [95]. It has been reported that the protective effect of adiponectin in chronic inflammation induced by DSS treatment [92]. The administration of adiponectin by adenovirus infection significantly reduces colitis severity in mice [127]. Moreover, adiponectin attenuates the stress signals and apoptotic status in colonic epithelial cells [128]. These findings support the concept that adiponectin derived from adipocytes plays an important role in maintaining intestinal homeostasis. In addition to adiponectin, apelin promotes the proliferation of epithelial cells in intestinal tissues and also plays a critical role in stabilizing the development of lymphatic vessels [114]. The administration of apelin can enhance lymphatic function of intestinal tissues to ameliorate the progress of colitis in IL-10 deficient mice [129]. These results imply that adiponectin and apelin may serve as therapeutic targets for patients with IBD. Thus, understanding the specific regulation between the inflammation of fat and intestinal tissues will become even more critical for IBD treatment in the foreseeable future. In addition, comparative effectiveness studies in CD revealed that pharmacological interventions for the adipokine TNF (infliximab) is correlated with the rates of IBD-associated hospitalization [130]. Population pharmacokinetic research for biologic agents in IBD, such as anti-TNF agents (infliximab) and anti-integrin agents (vedolizumab), has recognized high body weight as a risk factor correlated with the augmented drug clearance [131,132], suggesting that augmented levels of adipose-produced TNF confiscate anti-TNF agents. Moreover, similar results have been observed in other autoimmune disease patients with the treatment of obesity-related biologic agents [133,134]. These results suggest that obesity seems to enhance rapid clearance of biologic agents, and this correlation offers a chance for the treatment of obesity by pharmacological targeting on adipokines as additional approaches for patients with IBD.

Adipokines such as TNF and IL-6 can regulate immune homeostasis and affect the functions of immune cells [135]. Creeping fat is among the major sources of the proinflammatory cytokine IL-6 seen in IBD patients, and augmented circulating levels of IL-6 in diet-induced obese mice promote the development of insulin resistance and type 2 diabetes [136,137]. IL-6 plus TGF-b-driven Th17 cell differentiation promotes the expression of effector cytokines with inflammatory effects in the gut [9]. IL-6-mediated STAT3 activation via residues Tyr705 and Ser727 phosphorylation is also critical for IL-21-modulated differentiation of Th17 cells [8]. The axis of IL-6-STAT3 plays critical roles in the modulation of signaling between cytokines and cytokine receptors in the pathway to the progression of colitis [138]. Moreover, IL-6-STAT3-based and obesity-related metabolic disturbances enhance the development of immune cells to regulate obesity-associated inflammation and insulin resistance. These results provide a target for the treatment of obesity to metaflammation-linked pathologies of colitis and diabetes.

## 3. Conclusions

**Figure 3:**
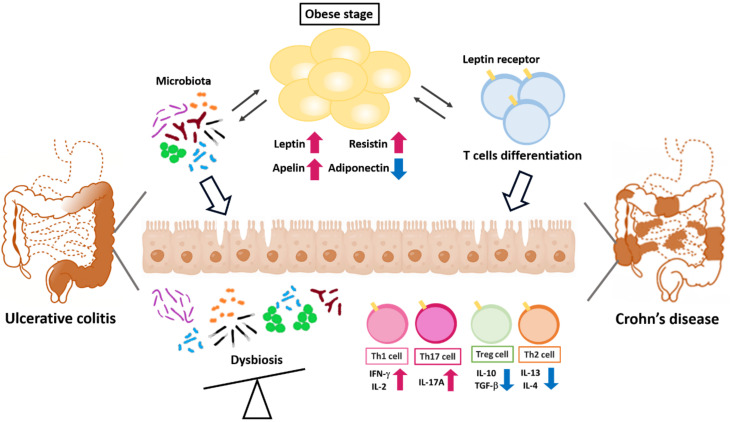
Adipokines regulate the polarity of T helper cells in adipose tissue and colons. Obesity-modulated chronic inflammation and gut microbiota dysregulation lead to the barrier integrity loss, allowing pathogenic T helper cell infiltration into the gut LP. Bacterial component translocation promotes a pro-inflammatory response by enhancing Th1 and Th17 cell differentiation.

This review has summarized observations that have contributed to the better understanding of the critical roles of adipokines in maintaining the immune homeostasis between macrophages and T helper cells during the development of inflammatory colon disease (Figure 3). Given the complexity of the function of adipokines, future work that aims to clarify the effects of these obesity-associated mediators in colon disease could focus on the following points: systematically analyzing the expression profiles of lineage-specific transcription factors for adipokine production in immune cells from dissimilar clinical samples and correlating these profiles with the stages of colon disease; characterizing the T-cell phenotypes of CD or UC patients, especially with respect to colon-infiltrating lymphocytes; and exploring the underlying mechanism of the alterations of adipokines in colonic tissues. Overall, studies of adipokine-mediated modifications in immune cells and the identification of associated immune disorders have provided important aspects of this regulatory machinery, and reveal new insights into immune regulation and the progress of therapeutic treatments targeting adipokines with specific roles in immune homeostasis rather than using broadly immunosuppressive agents. Therefore, adipokine-modulated pro-inflammatory cytokines may be a therapeutic target in inflammatory colon diseases.
